# Self-assembly of diphenylalanine peptide with controlled polarization for power generation

**DOI:** 10.1038/ncomms13566

**Published:** 2016-11-18

**Authors:** Vu Nguyen, Ren Zhu, Kory Jenkins, Rusen Yang

**Affiliations:** 1 Department of Mechanical Engineering, University of Minnesota, Minneapolis, MN 55455, USA

## Abstract

Peptides have attracted considerable attention due to their biocompatibility, functional molecular recognition and unique biological and electronic properties. The strong piezoelectricity in diphenylalanine peptide expands its technological potential as a smart material. However, its random and unswitchable polarization has been the roadblock to fulfilling its potential and hence the demonstration of a piezoelectric device remains lacking. Here we show the control of polarization with an electric field applied during the peptide self-assembly process. Uniform polarization is obtained in two opposite directions with an effective piezoelectric constant *d*_33_ reaching 17.9 pm V^−1^. We demonstrate the power generation with a peptide-based power generator that produces an open-circuit voltage of 1.4 V and a power density of 3.3 nW cm^−2^. Devices enabled by peptides with controlled piezoelectricity provide a renewable and biocompatible energy source for biomedical applications and open up a portal to the next generation of multi-functional electronics compatible with human tissue.

## Abstract (web summary)

Piezoelectricity in diphenylalanine peptide nanotubes (PNTs) suggests an avenue towards green piezoelectric devices. Here the authors show ‘smart' PNTs whose polarization can be controlled with an electric field, and a resultant power generator which harvests biomechanical energy with high power density.

## Graphical abstract

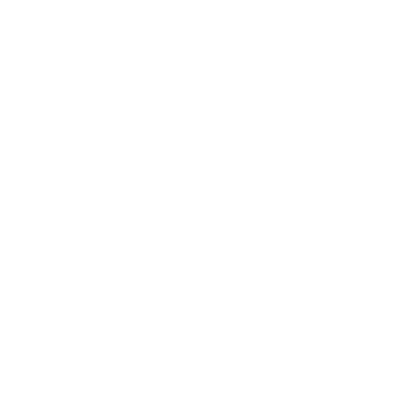

Piezoelectric phenomena have been widely observed in natural materials like bones^1^,^2^, collagen^3^,^4^, viruses^5^,^6^ and a variety of protein-based materials^7^. Although significant progress has been made toward the synthesis of piezoelectric biomaterials and their applications, challenges in aligning the polarization and improving the piezoelectricity continue to limit their applications. Various strategies have been developed to obtain alignment, such as molecular self-assembly^8^,^9^,^10^, template-assisted assembly^11^,^12^ and ferrofluid-assisted assembly^8^. However, most of the processes were relatively complex to scale up and could not be effectively used to achieve parallel polarizations. Piezoelectric devices based on biomaterials have also been demonstrated using viruses^5^, but their antiparallel polarizations and weak piezoelectric coefficients significantly limited their performance.

Among bio-inspired materials, peptides have emerged as a versatile class of materials thanks to their biocompatibility, functional molecular recognition and unique biological and electronic properties^13^,^14^,^15^,^16^,^17^,^18^,^19^. Diphenylalanine (FF), a short peptide consisting of two naturally occurring amino acid phenylalanine, has attracted increasing interest as the building block for ‘green' piezoelectric devices due to the exceptional piezoelectricity and remarkable mechanical properties of its derived structures^20^,^21^,^22^,^23^,^24^. Realizing macroscopic piezoelectricity in FF crystals requires uniform polarization with good alignment of their inherent electrical dipoles in the desired orientation. Like antiparallel dipole moments observed in M13 phages^5^, the oppositely directed polarizations were revealed in adjacent FF peptide nanostructures^20^,^25^. Although ferroelectric behaviour has been observed for FF nanotubes through high-temperature treatment, the polarization was only switched in the radial direction while the axial polarization was destroyed due to an irreversible phase transition to the centrosymmetric structure^26^. The high coercive field in the axial direction leaves native FF crystals practically unswitchable before electrical breakdown^27^.

Recently, our group has made an advancement in the alignment of FF structures by proposing a growth method through an engineered FF seed film^23^. By controlling water diffusion during the seed film formation, seeds with a majority of vertical domains with antiparallel polarizations could be achieved, and the application of an electric field made the polarizations uniform. The engineered seed film could also be used to grow microrods epitaxially. However, it was unclear if the vertical microrod array could be grown with controlled polarizations in two opposite directions, and a piezoelectric device was not realized.

Here we report the growth of FF peptide microrods with fully controlled polarization and improved piezoelectricity for fabricating a power generator. The outputs of the FF peptide-based generators are shown to exceed other bio-inspired generators, and are comparable to, or even better than, some generators based on inorganic materials. Our work makes a significant step forward in developing bio-inspired materials for piezoelectric devices and beyond.

## Results

### Growth of vertical FF microrods with controlled polarization

**Figure 1:**
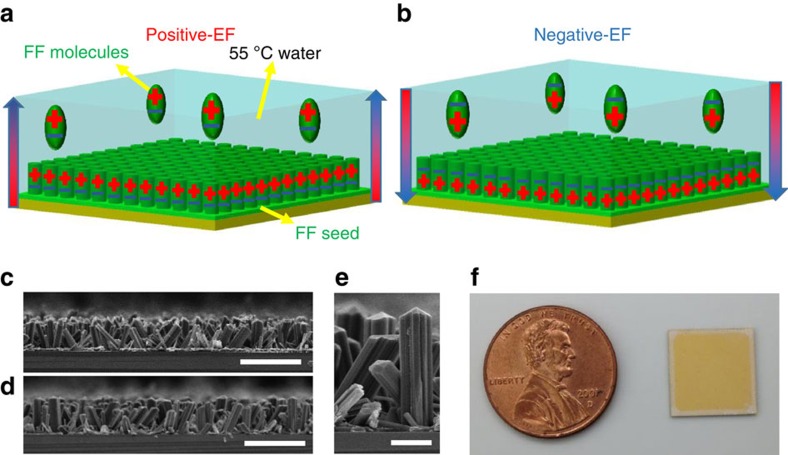
Growth of vertical FF peptide microrod arrays with controlled polarization. (**a**,**b**) Schematic of the positive-EF (electric field) growth (**a**) and the negative-EF growth (**b**). The large arrows are the directions of the applied electric fields, and the plus and minus signs indicate the polarizations of the FF molecules and FF microrods. (**c**,**d**) Cross-section views of arrays from the positive-EF growth (**c**) and the negative-EF growth (**d**). Scale bars in (**c**) and (**d**) are 100 μm. (**e**) High-magnification view of vertical microrods. Scale bar, 20 μm. (**f**) Photography of vertical FF peptide microrod array grown on a gold-coated substrate. The yellow gold layer is visible owing to the vertical alignment of microrod arrays and good optical waveguide properties along their axial directions.

We grew vertical FF peptide microrods from a substrate coated with a seed film in an FF-concentrated water solution^23^. An electric field was applied either along or opposite to the substrate surface normal, and the corresponding growth processes were called positive-EF (electric field) growth and negative-EF growth, respectively, (Fig. 1a,b). The mobile FF molecules in the solution were aligned so that their electrical dipoles were along the applied electric field during the FF peptide self-assembly^28^. The orientation of the FF molecules being deposited onto the seed determined the polarization of the microrods. Thus, the polarizations of the microrods were in the same direction as the applied electric field, and the spontaneous polarization should result in surface charges on the tips of microrods. Meanwhile, electric fields did not significantly affect the morphology of the microrods, as shown by scanning electron microscopy images (Fig. 1c–e and Supplementary Fig. 1a,b). Figure 1f shows uniform FF peptide microrod arrays grown on a 1.25 × 1.25 cm^2^ gold-coated silicon substrate. Since FF peptide microrods are a good optical waveguide axially but poor light transmitter radially^29^, the visibility of the yellow gold film provides an easy way to confirm the good vertical alignment of the microrod arrays (Fig. 1f and Supplementary Fig. 1c). The X-ray diffraction spectra of FF peptides revealed a hexagonal crystal structure with the space group P6_1_, allowing for a strong piezoelectric effect (Supplementary Fig. 2).

### Characterization of the polarizations of FF microrod arrays

**Figure 2:**
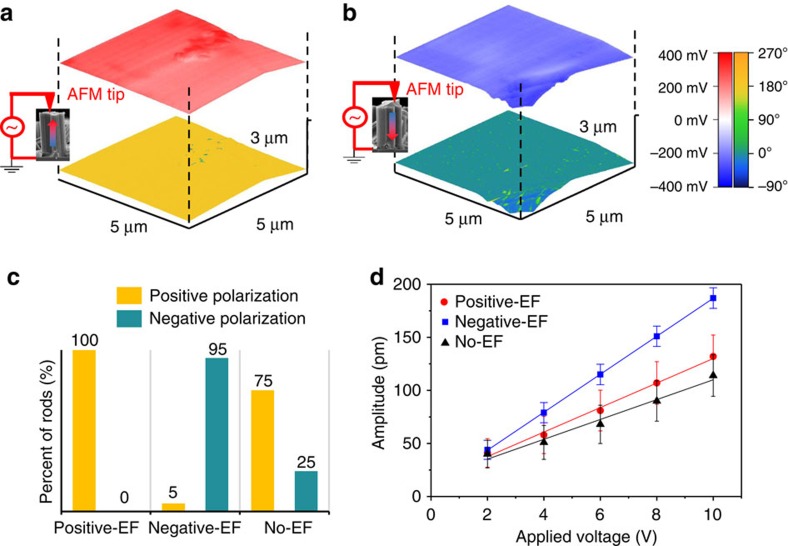
PFM and SKPM characterization of the microrod arrays. (**a**,**b**) PFM phase image and corresponding SKPM surface potential map of a microrod from the positive-EF growth (**a**) and a microrod from the negative-EF growth (**b**). The phase and surface potential distributions are shown by the colour overlaid on the topography of the top of the microrod. (**c**) Statistics of the piezoelectric phase for the arrays from the positive-EF growth, negative-EF growth and no-EF growth. Detailed data for this chart is provided in Supplemental Table 1. (**d**) Linear dependence of the PFM amplitude on the applied voltage for FF peptide microrods from growth with different electric fields. The slopes of the lines provide effective piezoelectric coefficients *d*_33_, which are 17.9, 11.7 and 9.3 pm V^−1^ for microrods from the negative-EF growth, positive-EF growth and no-EF growth, respectively. Error bar: s.d.

Piezoresponse force microscopy (PFM) was employed to investigate both the orientation of electrical polarization and the piezoelectric strength of FF peptide microrods. As we applied an alternating voltage on the FF peptide microrods, an FF peptide microrod with either upward or downward polarization deformed either out of phase (180°) or in phase (0°), respectively, (Supplementary Fig. 3). The phase distributions of vertical microrods from a positive-EF growth and from a negative-EF growth in Fig. 2a,b, respectively, show that applying opposite electric fields during growth results in FF peptides with phases of polarization 180° apart. Scanning Kelvin Probe Microscopy (SKPM) was also employed and confirmed that the surface charge on the microrod tip was consistent with the polarization, verifying the mechanism shown in Fig. 1a,b. The polarization was very stable and could not be switched with a high electric field once the growth was completed (Supplementary Fig. 4). Randomly selected 20 microrods from each growth process were tested (Fig. 2c and Supplementary Table 1). The results indicated that 95–100% of microrods had polarization in the direction of the applied electric field. In the absence of an electric field during growth, the polarization became much less uniform (Fig. 2c). A preferential polarization was often observed in such no-EF growth, which might have been due to native charges on the substrate or even small electric fields from surrounding equipment^28^,^30^,^31^. The inherent polarization of FF peptides originates from the ordered arrangement of positively charged amino termini (NH_3_^+^) and negatively charged carboxyl termini (COO^−^)^30^. Therefore, microrod arrays with opposite polarizations have either NH_3_^+^ groups or COO^−^ groups predominantly exposed at the top surface. Hence, the biofunctionality of the FF peptide microrod array can be tuned by simply switching the electric field during growth.

### Piezoelectric coefficient *d*_33_ of the FF microrod arrays

We examined the piezoelectric strength of vertical microrod arrays by measuring their effective piezoelectric constant *d*_33_ (Fig. 2d). The highest effective piezoelectric constant *d*_33_=17.9 pm V^−1^ was observed from this study, much higher than the previously reported value of 9.9 pm V^−1^ (ref. 23). The improved piezoelectricity is ascribed to a better alignment of FF peptide molecular dipoles assisted by the electric field and elevated temperature. Our result is consistent with a recent theoretical study, which shows that an external electric field stretches the peptide backbone of the FF molecule and significantly increases the dipole magnitude^28^. The temperature determines the number of water molecules in an FF peptide hexagonal crystal, and hence, the lattice constants and piezoelectricity^26^,^27^,^32^. However, further investigation on the effect of the electric field and temperature is needed to provide more insights.

### Characterization of power generation

**Figure 3:**
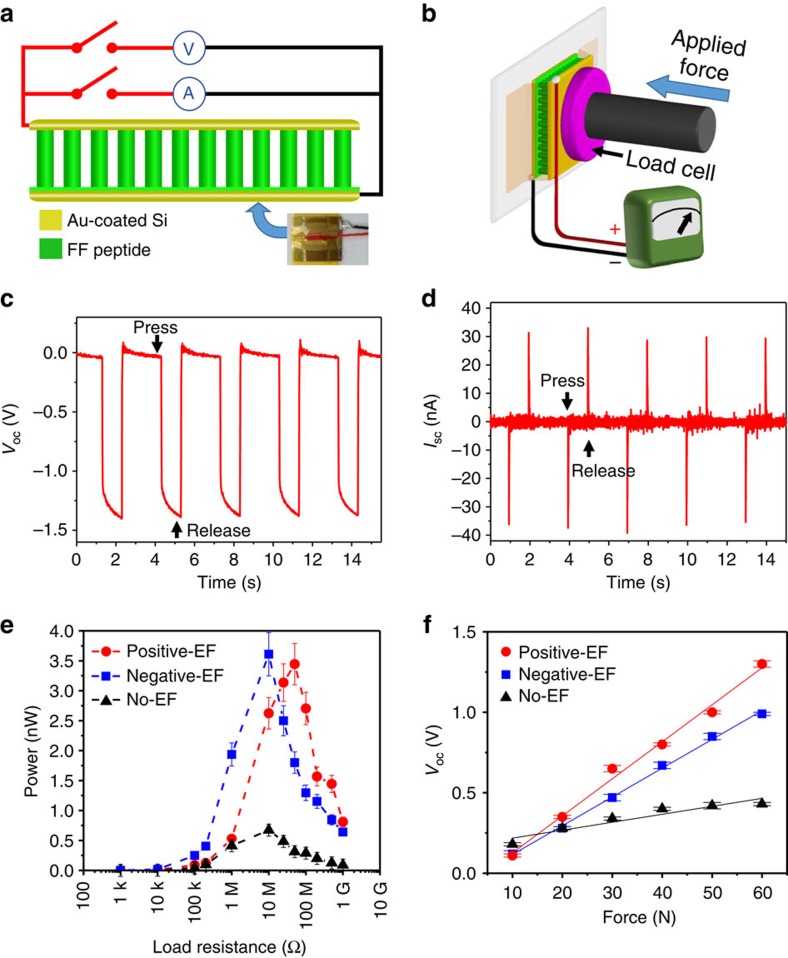
Characterization of the FF peptide-based power generators. (**a**) Schematic of the FF peptide-based generator connected to the measurement equipment. Bottom-right inset: photography of a real device. (**b**) Schematic of the measurement set-up in which a linear motor pushes with controlled forces on the top electrode in (**a**). The linear motor was programmed to always keep contact with the top electrode to minimize the effect of static charges. (**c**,**d**) Open-circuit voltage (**c**) and short-circuit current (**d**) from a generator using microrods from positive-EF growth. (**e**) Dependence of the power output from the generators on the resistance of the external load under 50 N applied force. (**f**) Linear dependence of the open-circuit voltage on the applied force. Error bar: s.d.

We investigated the power generation using FF peptide microrods with controlled polarizations. The FF peptide microrod array was sandwiched between two electrodes that connected to an external load or measuring instruments, as shown in Fig. 3a. When the device was compressed and released, FF peptide microrods converted the mechanical energy into electricity. The device characterization set-up in Fig. 3b includes a closed-loop linear motor with a load cell. A periodic compressive force was applied on the power generator for 1 s and released for 2 s (Fig. 3b). The power generation of FF peptide microrods from positive-EF growth is shown in Fig. 3c,d. Under an applied force *F*=60 N, the output open-circuit voltage (*V*_oc_) and short-circuit current (*I*_sc_) reached 1.4 V and 39.2 nA, respectively. FF peptide microrods from negative-EF growth produced opposite voltage and current outputs, and FF peptide microrods from no-EF growth produced much smaller output (Supplementary Fig. 5). The signs of open-circuit voltage and short-circuit current under compressing force all agree with the polarization direction measured from PFM. Reversed connection tests show that all outputs are reversed (Supplementary Fig. 6). The switching-connection test excluded the errors from the variation of contact resistance or parasitic capacitance and confirmed that the detected electrical signal was truly from the piezoelectric FF peptide microrods. Unlike generators based on virus or zinc oxide nanostructures, which showed rapid decay of the open-circuit voltage due to partially the leakage current through these materials^5^,^6^,^33^, the open-circuit voltage herein remained at a constant level owing to the excellent dielectric properties of FF peptides^34^ (Supplementary Fig. 7). When connected to external resistors, FF peptide microrods grown with electric fields produced up to 3.3 nW cm^−2^ at 50 MΩ, which is 3.8 times higher than the power density of zinc oxide nanowire-based generators (0.854 nW cm^−2^ for a single generator) driven by a 24 times greater pressure^33^. Our comparison here is conservative since the reported vertical zinc oxide nanowire-based generator was not connected to a load, so its power output was estimated using separately measured peak voltage (96 mV) and current (8.9 nA cm^−2^), which cannot be achieved simultaneously. In contrast, FF peptide microrods grown without electric fields yield power about 5 times lower than the power produced from microrods grown with electric fields (Fig. 3e), demonstrating the importance of the applied electric field for output enhancement. The effect of strain rate on the output of FF peptide-based generators was also investigated, and increasing strain rate was found to increase the peak power output up to 7 nW cm^−2^ (Supplementary Fig. 5e), which was comparable to the performance of a generator based on lead zirconate titanate nanoribbons^35^.

Time integration of current peaks yields an average charge *Q*=530 pC (Supplementary Table 2 and Supplementary Fig. 8). In the quasi-static case, the relation *d*_33_=*Q/F* holds^36^, from which we obtain an estimated piezoelectric constant *d*_33_≈8.8 pm V^−1^. It is smaller than the effective piezoelectric constant measured by PFM, indicating that not all microrods contribute to the power generation and that the device can be further optimized. The linear piezoelectricity of FF peptides was verified by the linear dependence of the *V*_oc_ and *I*_sc_ on the applied force (Fig. 3f and Supplementary Fig. 5). We performed control experiments by replacing the FF peptide microrod array with a non-piezoelectric Kapton thin film. The output signals became negligible (Supplementary Fig. 9), further validating the output of the generators.

### Stability and energy conversion of FF peptide generator

**Figure 4:**
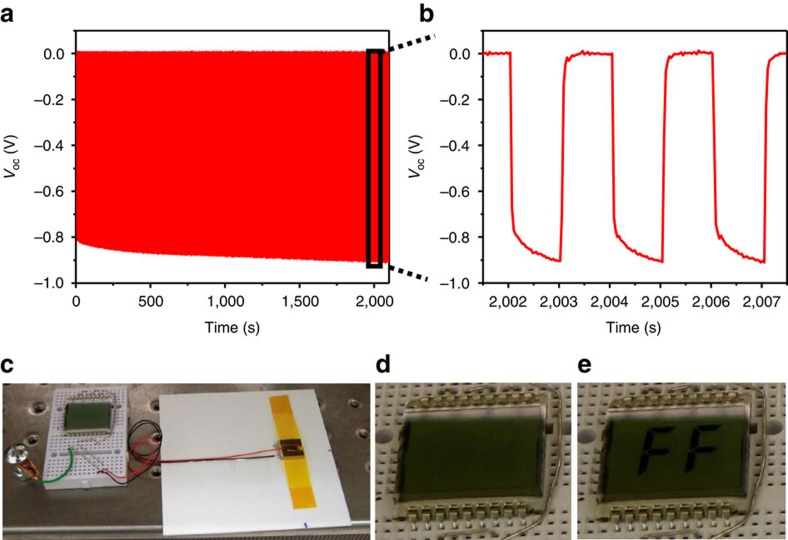
Demonstration of the FF peptide-based generator as a practical power source. (**a**,**b**) Open-circuit voltage over time as the generator was pressed under 50 N force for over half an hour at 0.5 Hz (**a**), and the enlarged view of the voltage output (**b**). The time was limited by the storage of our measuring instrument. The background shift due to long time measurement was subtracted from the recorded signal for clarity. (**c**) Photograph of the generator as a direct power source for an LCD. (**d**,**e**) Photograph of the LCD before (**d**) and after (**e**) the generator in (**c**) was pressed by a human finger.

The fatigue behaviour of the FF peptide-based generator was evaluated. We tested the *V*_oc_ of a generator under a cyclic force (50 N) for an extended period of time (Fig. 4a,b). The output voltage showed no degradation over more than 1,000 press/release cycles for more than half an hour. We repeated this test five times on the same device for a total of over 5,000 cycles and the device did not show any degradation, indicating the high durability of FF peptide-based devices. Finally, by stacking three power generators on top of each other, we were able to further increase the power output and directly drive a seven-segment liquid crystal display (LCD). The letters of ‘FF' on the LCD were activated when the generator was pressed by a human finger (Fig. 4c–e and Supplementary Movie 1 for an FF-based generator, Supplementary Movie 2 for a control device), demonstrating the capability of the FF-based power generator to collect biomechanical energy.

## Discussion

We have obtained vertical FF peptide microrods with controlled inherent polarization and improved piezoelectric strength. The microrod arrays are used to fabricate a power generator whose power density substantially exceeds that of similar devices. The output can be increased with stacked generators and an LCD could be driven simply by pressing the device with a finger. The uniform polarization in two opposite directions promises to expand the applications of FF peptide beyond energy harvesting and piezoelectric devices. Our present study on FF peptide power generators makes a significant step toward developing FF peptide into a smart, versatile and biocompatible material platform.

## Methods

### Fabrication of FF peptide microrod arrays

In positive-EF, negative-EF and no-EF growth, positive, negative and no electric fields were applied, respectively, during the self-assembly of FF peptides. The voltage was provided across two aluminum plates (15 × 15 cm^2^) by a high-voltage generator (Glassman High Voltage, Inc., model PS/MJ1OP1500-11). Five kilovolts across a 5 mm gap and 8 kV across a 4 cm gap were used in seed fabrication and solution growth, respectively. The seed film was prepared by drying a 20 μl drop of 1,1,1,3,3,3-hexafluoro-2-propanol (Sigma-Aldrich) with FF peptide (Bachem) at a concentration of 45 mg ml^−1^ on a 1.25 × 1.25 cm^2^ gold-coated silicon substrate. The stock materials were all stored in enclosed containers to minimize undesired water absorption from the environment. The obtained amorphous film was crystallized into vertical microrod domains by vigorously circulating 100% humid air for 60 s. Then an FF-water solution was prepared by dissolving FF peptide in water to 2 g l^−1^ concentration at 70 °C. The dissolution process was monitored so that the resulting solution was completely clear. Five millilitres of FF-water solution was then distributed into a 40 mm diameter Petri dish, which was placed between the two 15 × 15 cm^2^ aluminum plates, in an oven at 55 °C. The substrate with seed film was put upside down floating on the FF-water surface. The Petri dish was covered by a lid with five 5 mm diameter holes that limited the water evaporation rate and suppressed spontaneous nucleation in the FF-water solution. After 6 h of growth, the substrate was taken out and the residual water was blown off with compressed air.

### PFM and SKPM

PFM and SKPM were conducted on the top of the FF peptide microrods with an Asylum MFP 3D system. The probe used in both modes was an Asylum Research ASYELEC-01 with Ti/Ir coating on both cantilever and tip. The nominal resonance frequency of the probe was 70 kHz, and the tip radius was 28±10 nm according to the manufacturer. The inverse optical lever sensitivity and spring constant were calibrated before all measurements using Asylum's software GetReal. To determine the phase responses of the array, Dual AC Resonance Tracking mode was used to reduce noise and topography crosstalk. The probe was excited at 1 V amplitude and contact resonance frequency was typically ∼300 kHz. Correction for the instrumental phase offset was done by shifting the phase responses so that the phase at contact resonance was 90°. The tested 20 microrods were randomly selected at four 1 × 1 mm^2^ sites on the substrate, and were at least 200 μm apart. To determine the effective piezoelectric coefficient *d*_33_, Single frequency mode was used and the probe was excited at 20 kHz, which is far from contact resonance to avoid signal amplification. An area of 500 × 500 nm^2^ was scanned under AC voltages between 2 and 10 V. The average vibration amplitudes in each scanned area were recorded to calculate the effective *d*_33_. In all PFM scans, the probe was typically pressed on the sample surface with a force of about 100 nN. Surface potential was measured in SKPM mode using the dual-pass technique. In the first pass for detecting topography, the set point was 65–70% of free amplitude (∼100 nm), and the tip was lifted 50 nm above the surface in the second pass for measuring surface potential.

### Fabrication of FF peptide-based power generator

The substrate with FF peptide microrod arrays was fixed to a 10 × 10 cm^2^ alumina plate for convenient handling. A bare substrate coated with gold was held on top of the FF peptide microrod array by Kapton tape to serve as a top electrode. Two lead wires were connected to two gold electrodes using silver paste to complete the device fabrication.

### Characterization of the generators

Devices from all polarization scenarios were systematically measured, in which at least five cycles were run for each voltage, current and power data point before average values and errors were calculated. The FF-based power generator was mounted vertically to a fixture, and a horizontal force was applied by a linear motor (E100-RD-HC type with Force Control, LinMot) with a load cell (FC2231, Digi-Key) mounted on the tip. A minimum (5 N) force was maintained during the test to avoid creating an unintentional triboelectric effect due to the separation of the generator and the actuator. Thus, the applied force was the difference between 5 N and the peak force. The outputs of the generator were recorded using an electrometre (Keithley 6517B) and a current amplifier (Keithley 428). The generator was characterized in a Faraday cage to avoid interference from the surrounding equipment, and the linear motor was placed outside of the cage. For demonstration, a two-digit seven-segment LCD (Varitronix VI-201-DP-RC-S, Digi-Key) was wired according to the data sheet provided by the manufacturer to display the letters ‘FF'.

### Data availability

The data that support the findings of this study are available on request from the corresponding authors (R.Y.).

## Additional information

**How to cite this article:** Nguyen, V. *et al*. Self-assembly of diphenylalanine peptide with controlled polarization for power generation. *Nat. Commun.* **7,** 13566 doi: 10.1038/ncomms13566 (2016).

**Publisher's note:** Springer Nature remains neutral with regard to jurisdictional claims in published maps and institutional affiliations.

## Supplementary Material

Supplementary Information Supplementary Figures 1-9 and Supplementary Tables 1-2

Supplementary Movie 1 Control device with non-piezoelectric material produces no power and cannot activate an LCD.

Supplementary Movie 2 The peptide generator produces electricity as it is pressed by a human's finger, and the power is high enough to activate an LCD.
