# Supplementary material for: Self-assembly of diphenylalanine peptide with controlled polarization for power generation
Source: Nat Commun. 2016 Nov 18;7:13566. doi: 10.1038/ncomms13566 (PMC5120215; doi:10.1038/ncomms13566)
Supplement: Supplementary Information — Supplementary Figures 1-9 and Supplementary Tables 1-2 [file ncomms13566-s1.pdf]

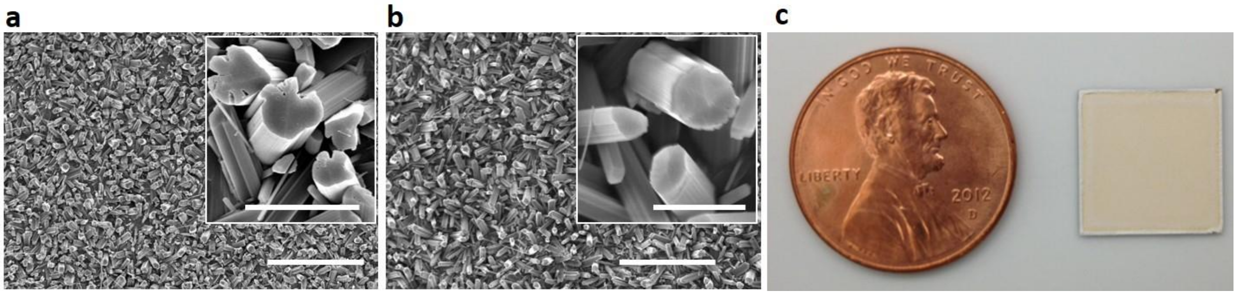

Supplementary Figure 1| Vertical FF peptide microrod arrays. **a,b,** Top view of the microrod arrays from the positive-EF growth (a) and negative-EF growth (b). Insets: enlarged view of microrods. The scale bars in low-magnification images are 200 µm, and the scale bars in insets are 20 µm. **c**, A gold-coated substrate with mostly horizontal peptide microrods. The gold layer electrode is hardly visible owing to the poor light transmission in the radial direction of FF peptide microrods.

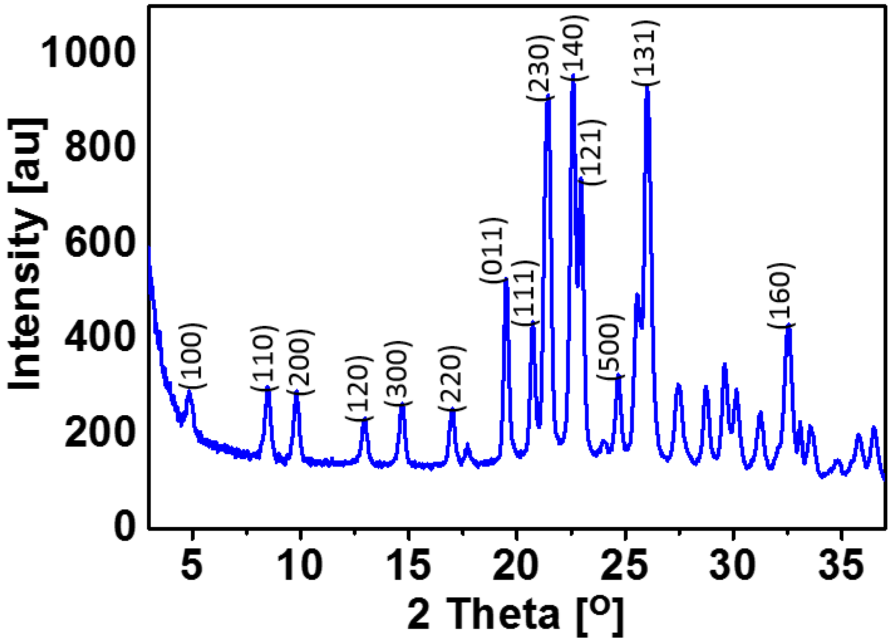

Supplementary Figure 2| X-ray diffraction (XRD) spectra of FF peptide microrods. XRD confirms that the FF peptide microrod arrays possess the hexagonal crystal structure with the space group $P6_1$ (noncentrosymmetric), allowing for a strong piezoelectric effect. Measurement was made using Bruker D8 system with Cobalt anode (λ=1.78899Å)

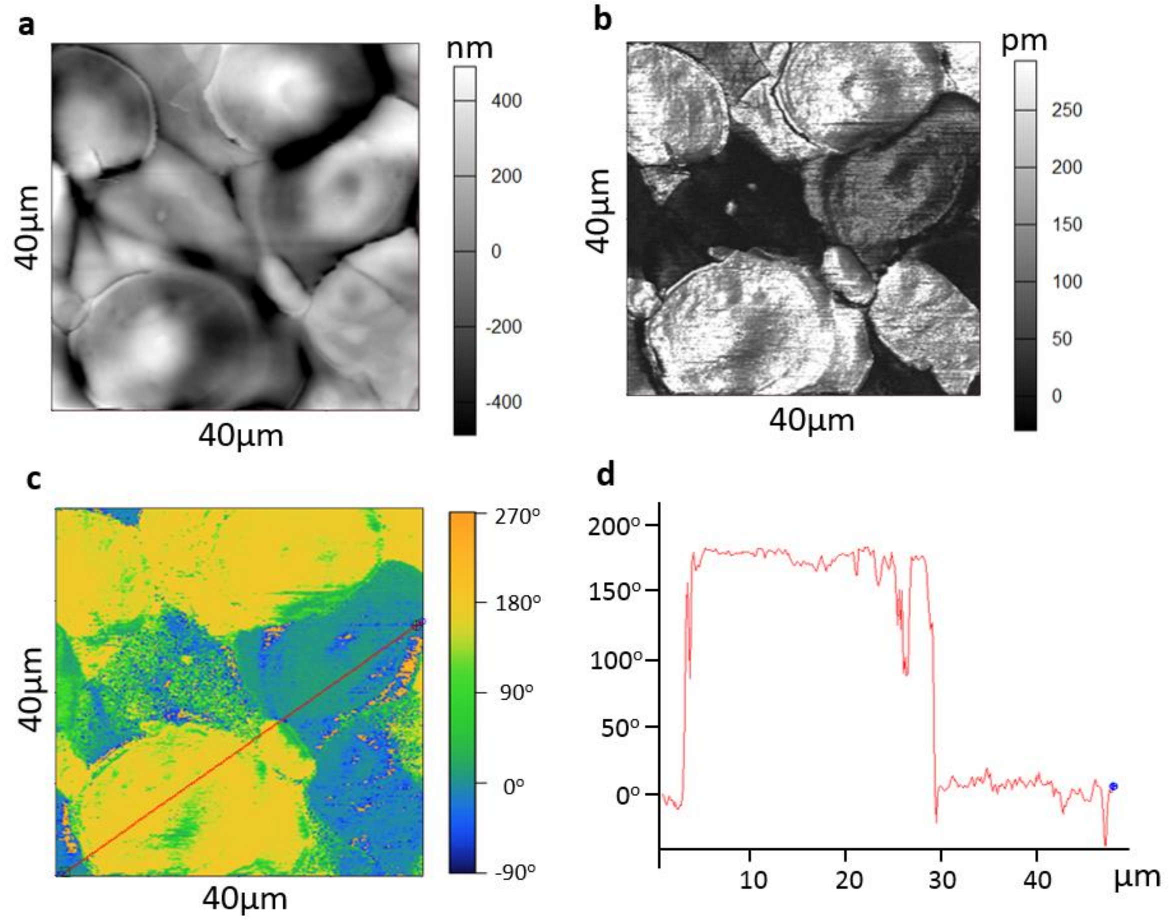

Supplementary Figure 3| PFM measurements of FF peptide crystals. FF peptide crystals in a seed layer grown with no electric field, showing both positive and negative polarities with 180° phase difference. **a,b,c,** Topography (a), amplitude (b) and phase response (c) of FF peptide crystals with opposite polarities. **d**, cross section at the red line in the phase map in (c), showing that the two polarities are approximately 180° apart.

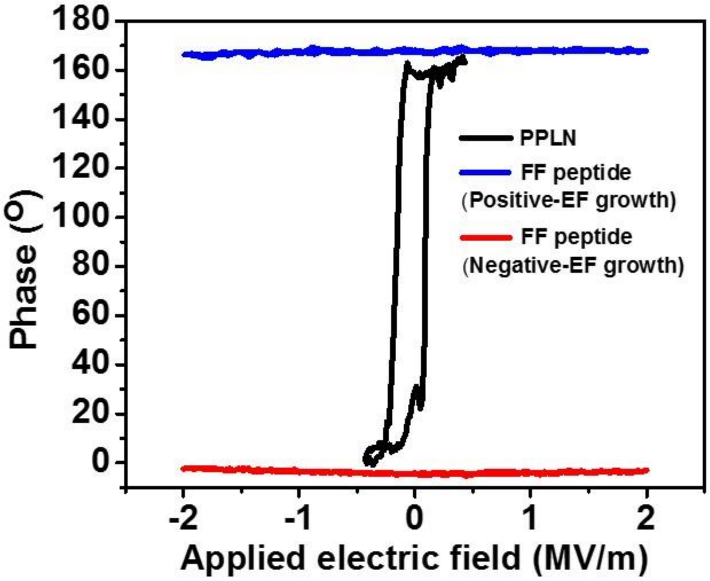

Supplementary Figure 4| Hysteresis loop measurements of FF peptide microrods. The control sample is a standard periodically poled lithium niobate (PPLN, Asylum Research). Compared to the PPLN, FF peptides have a much higher coercive field. Both the positive and negative polarizations were confirmed to be stable under high electric field. The polarizations could not be switched before the material was destroyed from electrical breakdown.

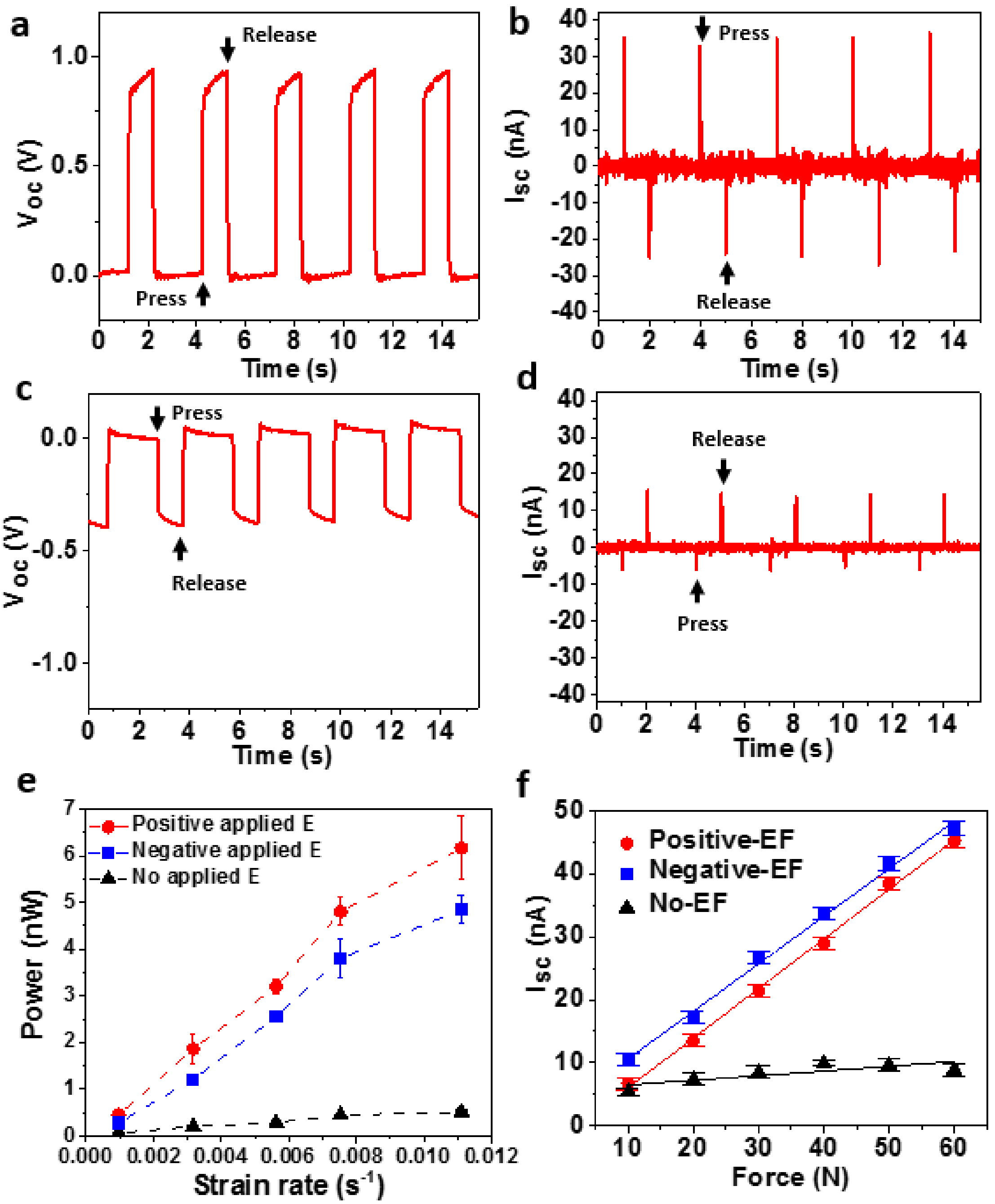

Supplementary Figure 5| Characterization of the FF peptide-based power generators. **a,b,c,d**, open-circuit voltage (a,c) and short-circuit current (b,d) of generators using microrods from negative-EF growth (a,b) and no-EF growth (c,d). **e**, dependence of peak output power on strain rate at 50 N applied force. The peak strain of 4.5×10^{-4} was estimated using a microrod array with 34,000 rods cm^{-2}, 15 µm-diameter, 50 µm-length, and 19 GPa Young's Modulus for each rod, and the strain was reached in a time that was equal to the force rise time (460, 140, 79, 59, 40 ms). **f**, dependence of peak short-circuit current on applied force. Error bar: s.d.

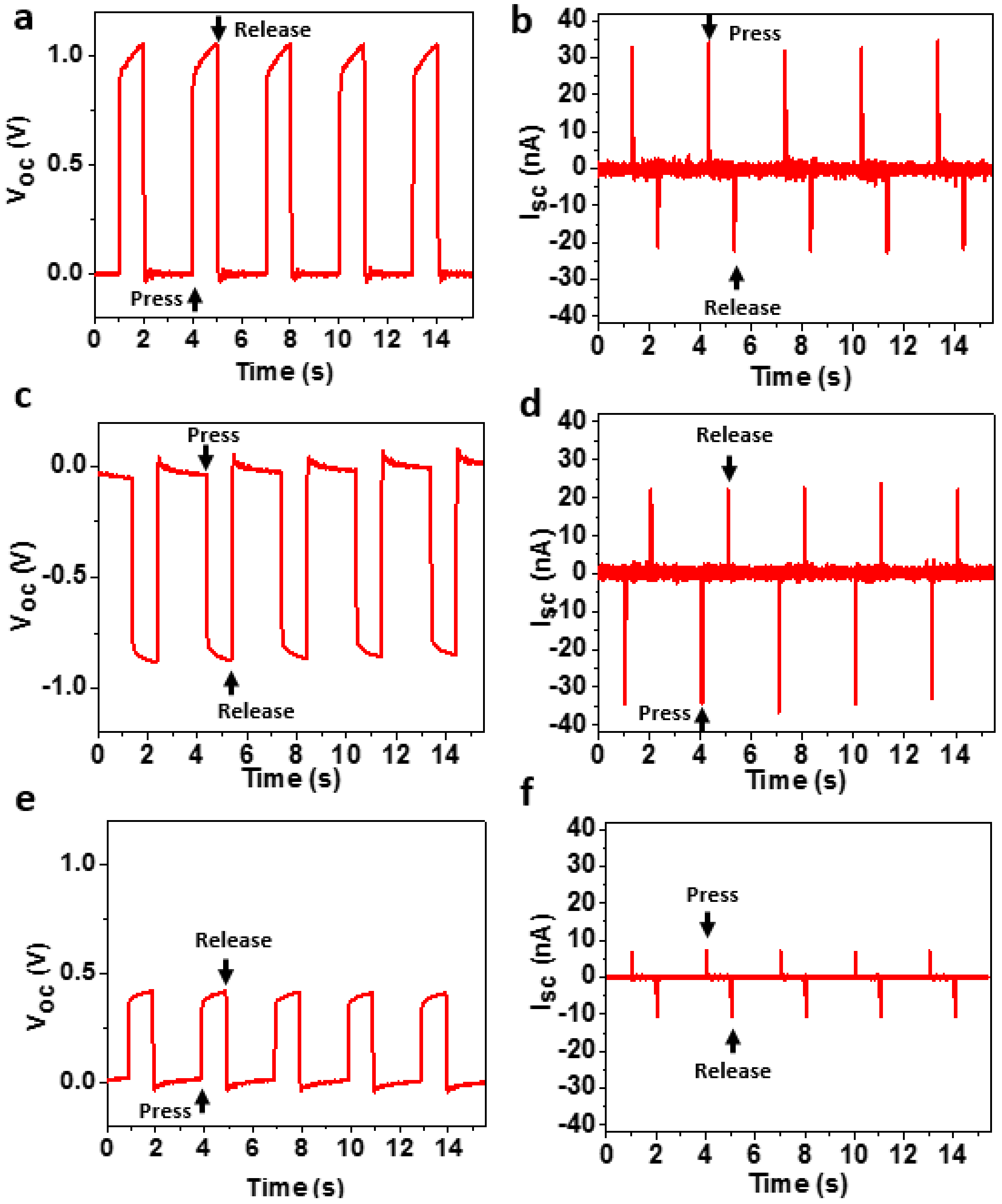

Supplementary Figure 6| Reversed connection test for FF peptide-based power generators. **a,b,c,d,e,f**, open-circuit voltage (a,c,e) and short-circuit current (b,d,f) from power generators using microrods from positive-EF growth (a,b), negative-EF growth (c,d), and no-EF growth (e,f) under reversed connection.

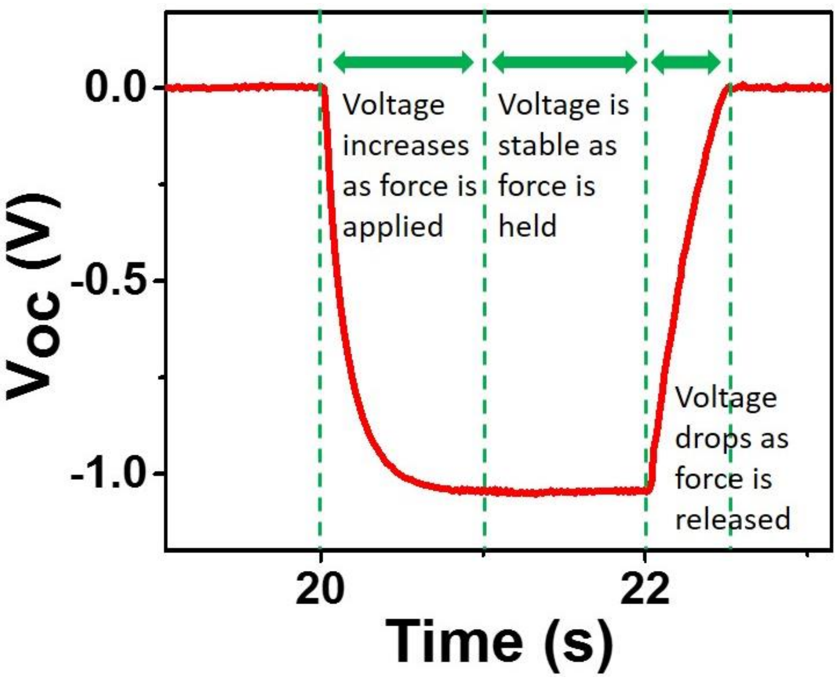

Supplementary Figure 7| Stability of the generated voltage over time. The constant voltage indicates extremely low leakage current, owing to the excellent dielectric property of FF microrods. The dwell time of applied force in Figure 3c,d was doubled here to confirm the negligible decay of the open-circuit voltage output.

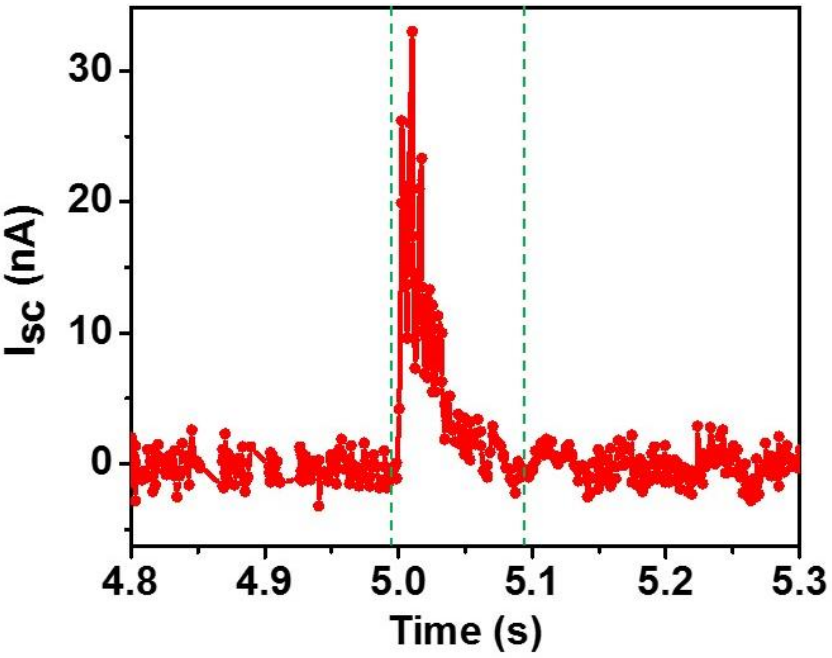

Supplementary Figure 8| Zoom-in view of a short-circuit current peak. The width of the current peak is about 100 ms. The current could be still generated after 100 ms but was not visible due to noise in the measurement system.

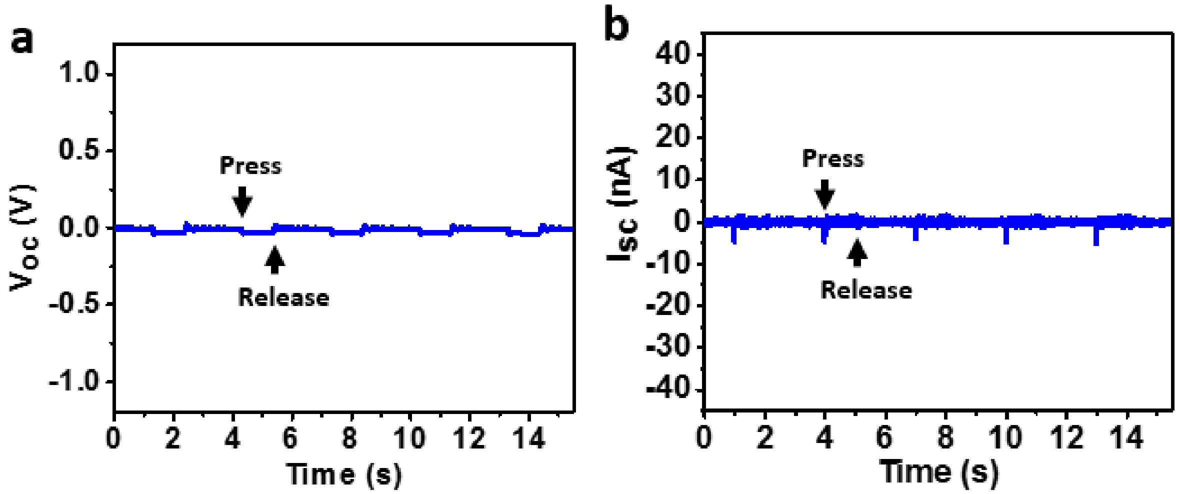

Supplementary Figure 9| Power output control measurement. **a,b**, Open circuit voltage (a) and short-circuit current (b) of a control device in which the FF peptide microrod array was replaced by a 50 µm-thick non-piezoelectric Kapton film.

Rod #	Phase response (degree)		
	Positive EF microrod	Negative EF microrod	No EF microrod
1	184	17.5	10
2	183	13.5	191.5
3	182	8.5	183
4	183.5	6.5	181.5
5	183.5	5.5	17
6	183.5	5.5	6.5
7	183	5.5	183.5
8	187.5	5.5	183.5
9	183.5	7.5	184.5
10	182.5	10.5	180.5
11	200.5	5	182
12	191.5	11	186
13	191.5	9.5	181.5
14	183	9.5	8
15	183.5	12	9.5
16	184	6	184
17	185.5	8.5	183
18	182.5	185.5	184
19	179	6.5	184
20	183.5	7	183.5

Supplementary Table 1| Summary of phase responses of microrods from the positive-EF growth, negative-EF growth, and no-EF growth.

Peak #	Charge generated (pC)	Peak #	Charge generated (pC)
1	509	6	551
2	522	7	539
3	477	8	521
4	598	9	506
5	504	10	568
**Average**	**530 pC**		

Supplementary Table 2| Summary of generated charges calculated by the integration of short-circuit current peaks over time.
